# Mobilisation of neural structures opens up new possibilities for idiopathic scoliosis treatment

**DOI:** 10.1186/1748-7161-7-S1-O56

**Published:** 2012-01-27

**Authors:** E Santos Leal

**Affiliations:** 1 Pysiotherapiecenter, Iserlohn, Germany

## Background

Idiopathic Scoliosis (IS) is a progressive disorder with reduced mobility of the spine. This makes corrections difficult or sometimes even inhibits postural changes. Even when correction of frontal or coronal plane deformity seems successful, sagittal correction mostly is not addressed appropriately. This might be due to functional tethering of the spinal cord or even of peripheral neural structures. This explains the lack of success when trying to correct sagittal plane deformity with the help of stretching exercises. Purpose of this study was to test how the release of these structures may relax lordotic tension in the thoracic area.

## Materials and methods

Finger – floor distance and sciatic slump was tested in 25 patients before and after a session of physiotherapy using neural tension exercises (Butler) [1,2].

## Results

Significant differences have been achieved with the help of these techniques (16 – 21 cm at the start independent of patient age / 3-0 cm at the end) with improved kyphosation at the same time.

## Conclusions

While in sole stretching of the hamstrings the sagittal profile cannot be improved, neurodynamics provide an improvement of this deformity. Neurodynamics therefore seem to be a useful add on in the physical treatment of IS.
